# Six Weeks of Daily Abaloparatide Treatment Increased Vertebral and Femoral Bone Mineral Density, Microarchitecture and Strength in Ovariectomized Osteopenic Rats

**DOI:** 10.1007/s00223-016-0171-1

**Published:** 2016-07-09

**Authors:** Hila Bahar, Kyla Gallacher, Julie Downall, Carol A. Nelson, Maysoun Shomali, Gary Hattersley

**Affiliations:** Radius Health, 950 Winter Street, Waltham, MA 02451 USA

**Keywords:** Abaloparatide, Anabolic treatment, PTHR1, PTH, PTHrP, Osteoporosis, Bone strength

## Abstract

Abaloparatide is a novel, potent and selective activator of parathyroid hormone receptor 1 (PTHR1) under clinical development for the treatment of osteoporosis. We assessed the effect of 6 weeks of abaloparatide on bone mass, microarchitecture, quality and strength in ovariectomized (OVX) rats. After 8 weeks of post-surgical bone depletion (baseline), OVX rats (*n* = 20–21/group) received daily subcutaneous vehicle (OVX-Veh) or abaloparatide at 5 or 20 µg/kg. Sham-operated control rats (*n* = 24) received vehicle. Areal bone mineral density (aBMD) of the lumbar spine (L4), total femur and femur diaphysis was measured at baseline and after 6 weeks of treatment. Femur and vertebral bone architecture and mechanical properties were assessed at the end of the treatment phase. At baseline, OVX-Veh rats exhibited significantly lower aBMD relative to Sham controls. Treatment of OVX rats with abaloparatide at 5 or 20 µg/kg/day increased aBMD dose-dependently in the lumbar spine, total femur and femur diaphysis to levels exceeding OVX-Veh or Sham controls. The abaloparatide 5 and 20 µg/kg groups had improved trabecular microarchitecture relative to OVX vehicle, with trabecular BV/TV exceeding OVX-Veh control values by 57 and 78 % (respectively) at the lumbar spine, and by 145 and 270 % at the distal femur. Femur diaphyseal cortical volume and thickness were significantly greater in the abaloparatide 20 µg/kg group relative to OVX vehicle or Sham controls. Bone strength parameters of the femur diaphysis, femur neck and L4 vertebra were significantly improved in the OVX-ABL groups relative to OVX-Veh controls. Bone mass–strength relationships and estimated intrinsic strength properties suggested maintained or improved bone quality with abaloparatide. These data demonstrate skeletal restoration via abaloparatide treatment of osteopenic OVX rats, in association with improved trabecular microarchitecture, cortical geometry and bone strength at sites that have clinical relevance in patients with osteoporosis.

## Introduction

Osteoporosis is a systemic skeletal disease characterized by low bone mass and microarchitectural deterioration of bone tissue with a consequent increase in bone fragility and susceptibility to fractures at multiple skeletal sites, most often at the spine, hip or wrist [1]. Osteoporosis is estimated to affect >20 million Americans, with 1.5 million osteoporotic fractures occurring in the USA every year [2]. As the population ages, the prevalence of osteoporosis and the incidence of osteoporotic fracture is increasing. Parathyroid hormone (PTH) and PTH(1-34) (teriparatide) are the only anabolic agents currently approved for treatment of osteoporosis. PTH is secreted by the parathyroid glands in response to low plasma calcium and acts directly on bone and kidneys to restore blood calcium levels [3]. PTH and PTH(1-34) were shown to be safe and effective in increasing bone mass and reducing fracture risk in postmenopausal women with osteoporosis [4, 5]. The anabolic mechanism of PTH and PTH(1-34) involves the stimulation of new bone formation, but this action is typically coupled by increased bone resorption activity [3, 6–8], and recent clinical data indicate that this resorptive response may significantly limit bone mineral density (BMD) gains achieved with intermittent PTH(1-34) therapy, notably at cortical sites [9]. Moreover, increased bone resorption with PTH(1-34) is associated with and may contribute to increase serum calcium levels [10, 11].

Signaling mediated via the PTH 1 receptor (PTHR1) plays an important role in skeletal development and homeostasis. Although both PTH and PTH-related peptide (PTHrP) act through the common PTHR1, mounting evidence suggests distinct skeletal roles for PTH and PTHrP. While both ligands can stimulate bone formation pharmacologically, PTHrP is uniquely involved in maintaining post-natal bone mass. For example, mice genetically deficient in PTHrP develop osteoporosis [12–14], whereas mice deficient in PTH exhibit increased bone mass that may be indirectly mediated by a local compensatory induction of PTHrP [13]. These findings suggest that PTHrP is a naturally occurring skeletal anabolic agent, the lack of which is not physiologically compensated by endogenous PTH [12–16]. Distinct receptor binding properties may be responsible for the differential effects of PTH and PTHrP [17–19]. Consistent with the genetic evidence for PTHrP as an important endogenous anabolic factor, a pharmacology study in adult ovariectomized (OVX) rats showed that 6 months of daily administration of native PTHrP(1-36) markedly enhanced bone mass and biomechanical properties [20]. Short**‐**term studies with limited numbers of human volunteers also indicate that treatment with native PTHrP(1-36) increased bone formation markers [21–24] and BMD [24], which has driven interest over the past several years in testing PTHrP(1-36) as a potential treatment for osteoporosis. The results of these studies have been mixed, with some suggesting that intermittent administration of high-dose native PTHrP(1-36) increases bone formation without concomitant stimulation of bone resorption and others reporting measurable stimulation of bone resorption and significant hypercalcemia [21–24].

Abaloparatide (ABL; previously referred to as BA058) is a novel 34 amino acid peptide selected for its potent and selective activation of PTHR1 signaling. Abaloparatide has 41 % homology to PTH(1-34) and 76 % homology to PTHrP(1-34) and was selected to retain potent bone anabolic activity but with a limited effect on bone resorption and low calcium-mobilizing potential [25]. In a complete Phase 2 clinical trial in postmenopausal woman with osteoporosis, daily subcutaneous injection of abaloparatide for 24 weeks increased BMD of the lumbar spine, total hip and femoral neck in a dose-dependent manner, in association with increased biochemical markers of bone formation. Moreover, the abaloparatide-induced BMD increases at the total hip were greater than achieved with teriparatide treatment [25]. These results are consistent with the bone anabolic activity and profile of abaloparatide.

The current work represents the first published nonclinical data on the effects of abaloparatide on bone. This study assessed the effects of 6-week daily administration of 2 doses levels of abaloparatide on bone in adult osteopenic OVX rats. The OVX rat model of postmenopausal osteoporosis allowed for high-resolution imaging of various skeletal sites to quantify microarchitectural and geometric changes in cancellous and cortical bone. Destructive ex vivo biomechanical testing was also performed to assess changes in the strength of the femur shaft, femur neck and lumbar vertebra. Results of the current study indicated a favorable efficacy and bone safety profile with 6 weeks of abaloparatide administration, which serves as a foundation for future bone quality, efficacy and safety studies of this novel agent.

## Materials and Methods

### Animals

All procedures, protocols and study designs were reviewed, approved and overseen by the Institutional Animal Care and Use Committee (IACUC) at Radius Health, Inc. A total of 93 ten-week-old virgin female Sprague–Dawley rats (Charles River Laboratories) were housed individually in ventilated, polycarbonate cages with access to food and water ad libitum. Their environment was maintained at 18–26 °C with 30–70 % relative humidity and a 12-h light/dark cycle. Animals were observed daily for clinical signs and weekly for changes in body weight.

### Experimental Design

After a 2-week acclimation period, animals were randomly allocated to ovariectomy or sham surgery groups. At 12 weeks of age, animals were either sham-operated (Sham) or ovariectomized (OVX) and remained untreated for 8 weeks (bone depletion period). The osteopenic OVX rats were then treated for 6 weeks by daily SC injection with either vehicle (0.9 % NaCl; OVX-Veh group; *n* = 21) or abaloparatide at 5 µg/kg (OVX-ABL5; *n* = 20) or 20 µg/kg (OVX-ABL20; *n* = 20). Sham rats received SC vehicle (*n* = 24). The number of animals per group was chosen to provide a robust preliminary assessment of bone safety, based on ex vivo bone strength assessments that have modest statistical power due to the absence of baseline control data. The study design is outlined in Table 1. Areal BMD (aBMD) of the total femur was measured in all groups by in vivo dual X-ray absorptiometry (DXA) at the end of the 8 weeks post-surgical bone depletion period. These data documented the negative impact of OVX on BMD and served as pre-treatment baseline values to evaluate percent change in BMD. aBMD of the total femur, femur diaphysis and lumbar vertebra was also assessed in the OVX-Veh, OVX-ABL5 and OVX-ABL20 groups at the end of the 6-week treatment period to evaluate treatment effects. All DXA measurements were performed by PixiMus (GE-Lunar Corporation, Fitchburg, WI, USA) on animals that were anesthetized with isoflurane. Animals were then euthanized, and the femurs and L4 vertebrae were collected, wrapped with ethanol-soaked gauze and frozen at −20 °C for high-resolution microcomputed tomography (µCT) and biomechanical testing. Successful OVX surgery was confirmed postmortem by assessing uterine atrophy and the absence of ovaries, and corroborated by DXA-based analysis of BMD changes, as described below.

**Table 1:** Study design

Surgical status	Treatment	*n*	Sex Species Age	Dosing regimen
Sham	Vehicle	24	Female Sprague–Dawley rats 20 weeks	6 weeks daily SC treatment
OVX	Vehicle	21		
OVX	ABL 5 µg/kg	20		
OVX	ABL 20 µg/kg	20		

*SC* subcutaneous, *ABL* abaloparatide, *Sham* sham-operated rats, *OVX* ovariectomized rats

### Microcomputed Tomography (µCT) Measurements

Quantitative µCT (mCT40 µCT scanner, Scanco Medical AG, Basserdorf, Switzerland) was used ex vivo to assess trabecular architecture in the fourth lumbar vertebra (L4) and the distal femoral metaphysis, and cortical bone geometry at the mid-femoral diaphysis, in accordance with recently published guidelines [26]. Scanning of trabecular bone at the distal femoral metaphysis was initiated distally at the level of the growth plate and extending proximally 250 slices. Evaluations were performed on 150 slices beginning from approximately 0.2 mm proximal to the growth plate. The entire L4 vertebra was scanned, and the trabecular bone between the cranial and caudal growth plates and the cortex was evaluated. Morphometric parameters were also evaluated, including bone volume fraction (BV/TV, %), bone volume (BV, mm^3^), total volume (bone plus marrow; TV, mm^3^), trabecular number (Tb.N, 1/mm), trabecular thickness (Tb.Th, mm), trabecular spacing (Tb.Sp, mm), connectivity density (Conn.D, 1/mm^3^), structural model index (SMI) and bone density (BD, mg/mm^2^). At the femoral mid-shaft (cortical bone), 23 transverse CT slices were obtained and used to compute cortical area (Ct.Ar), total area (Tt.Ar), Ct.Ar/Tt.Ar, marrow area (M.Ar) and cortical thickness (Cort.Th, mm).

### Biomechanical Testing

All biomechanical tests were performed using an Instron Mechanical Testing Instrument (Instron 4465 retrofitted to 5500). L4 vertebrae were tested in a destructive biomechanical compression test. Fresh-frozen vertebrae were thawed to room temperature, and the posterior pedicle arch, spinous process, and cranial and caudal ends were then removed to obtain a vertebral body specimen with two parallel surfaces and a height approximately equal to 4 mm. Width in the medial–lateral and anterior–posterior directions at both the cranial and caudal ends was measured for the calculation of cross-sectional area. The L4 bodies were placed between two platens, and a load was applied at a constant displacement rate of 6 mm/min until failure. Load–displacement curves were recorded by instrument software (Bluehill v2.5, Instron). The locations for determining maximum load at failure, stiffness and energy absorbed were identified manually from the load–displacement curve and calculated by instrument software (Bluehill v2.5, Instron). The intrinsic property ultimate strength was calculated from maximum load (N), cross-sectional area and height (mm) divided by BV/TV that was obtained by micro-CT. Prior to biomechanical testing, peripheral quantitative computed tomography (pQCT) was performed on the excised right femurs using a Stratec XCT-RM and associated software (Stratec Medizintechnik GmbH, Pforzheim, Germany; software version 5.40). Scans were performed at 50 % of the total femoral length from the distal end of the femur, corresponding to the mid-diaphyseal failure site expected for a three-point bending test. The position was verified using scout views, and one 0.5-mm slice perpendicular to the long axis of the femoral shaft was acquired. The scans were analyzed using a threshold for delineation of the external boundary. Axial area moment of inertia was calculated from the pQCT scan data and was used in the calculation of intrinsic strength parameters of the femoral shaft.

Femurs were then subjected to a destructive three-point bending test. Each whole right femur was placed on the lower supports of a three-point bending fixture with the anterior side facing down. The span between the two lower supports was set at 14 mm. The upper loading device was aligned to the center of the femoral shaft, and loading was applied at a constant displacement rate of 6 mm/min until failure. The locations for determining maximum load, stiffness and energy absorbed were identified manually from the load and displacement curve and values calculated by instrument software (Bluehill v2.5, Instron). The intrinsic properties ultimate strength, elastic modulus, and toughness were calculated from maximum load (N), stiffness (N/mm), energy absorbed (mJ), anterior–posterior diameter (mm) and moment of inertia (mm^4^). After three-point bending, the proximal half of the femur was retrieved for cantilever compression testing of the femoral neck. The proximal femur was placed firmly in an anchoring platform with the greater trochanter lodged into a notch in the platform. Loading was applied to the femoral head with a stainless steel probe, parallel to the femoral shaft at a constant displacement rate of 6 mm/min until failure. The locations for determining maximum load (N), stiffness (N/mm) and energy absorbed (mJ) were identified manually from the load and displacement curve and calculated by instrument software (Bluehill v2.5, Instron).

### Statistical Analysis

Results are expressed as mean and standard deviation. All statistical analyses were performed using ANOVA followed by Tukey’s multiple comparison test (GraphPad Instat, Cary, NC, USA; release 9.1, or GraphPad Prism, Cary, NC, USA; version 6.07). Linear regression analyses were conducted with GraphPad Prism, version 6.07.

## Results

### General Health

OVX was associated with an expected increase in body weight gain relative to ovary-intact Sham controls (data not shown). Daily cage-side observations did not identify any clinical signs of hypercalcemia, and abaloparatide did not impair body weight gain at either dose. The high dose of abaloparatide was associated with slightly greater body weight after 6 weeks of administration (mean ± SD = 414 ± 43 g) relative to OVX-Veh controls (398 ± 42 g). Abaloparatide was well tolerated at both dose levels for the duration of the study.

### Areal BMD (aBMD)

**Fig. 1:**
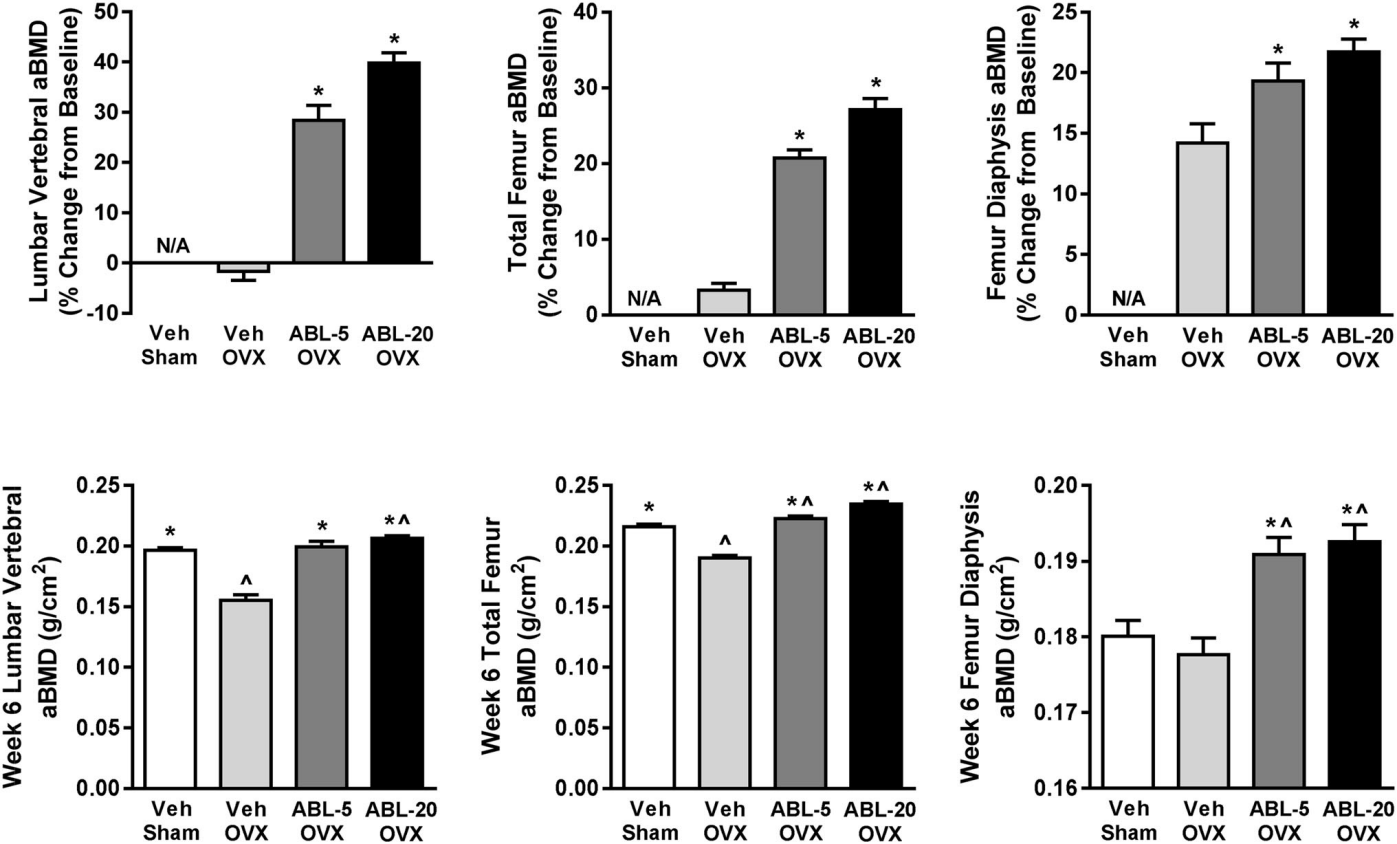
Effect of abaloparatide administration on aBMD in OVX rats. *Upper panels* indicate percent change from the pre-treatment baseline to the end of the 6-week treatment period. *Lower panels* indicate absolute aBMD values at the end of the 6-week treatment period. ABL-5, abaloparatide 5 µg/kg/day; ABL-20, abaloparatide 20 µg/kg/day. Data represent mean ± SEM, *n* = 20–24 per treatment group. *P < 0.001 versus OVX-Veh. ^^^ *P* < 0.001 versus Sham-Veh. N/A = not applicable due to lack of baseline data for the Sham-Veh group

Total femur aBMD was measured 8 weeks after surgery in order to assess OVX-induced bone depletion prior to treatment initiation. At this baseline, total femur aBMD was decreased by a significant 11 % for all 61 OVX rats combined (mean ± SD = 0.1845 ± 0.006 gm/cm^2^) compared to a subset of 10 randomly selected Sham controls (0.2045 ± 0.009 gm/cm^2^, *P* < 0.001). Total femur aBMD values in OVX-Veh controls rats remained decreased compared to intact Sham controls after 6 weeks of treatment (14 % decrease, *P* < 0.001) (Fig. 1).

aBMD was also measured at the total femur, femur diaphysis and lumbar spine in all groups after 6 weeks of daily treatment with vehicle or abaloparatide. Compared to pre-treatment baseline values, OVX rats treated with abaloparatide at 5 or 20 μg/kg exhibited significant increases in BMD at the spine (+27 and +39 %, respectively, *P* < 0.001; Fig. 1), while OVX-Veh controls showed modest bone loss. Increments in spine aBMD with abaloparatide resulted in final spine BMD values that significantly exceeded those of OVX-Veh controls (by 28 and 33 %, for OVX-ABL5 and OVX-ABL20 groups, respectively; *P* < 0.001, Fig. 1). Furthermore, post-treatment spine aBMD for the OVX-ABL20 group significantly exceeds that of ovary-intact Sham control animals (*P* < 0.001 vs. Sham).

**Fig. 2:**
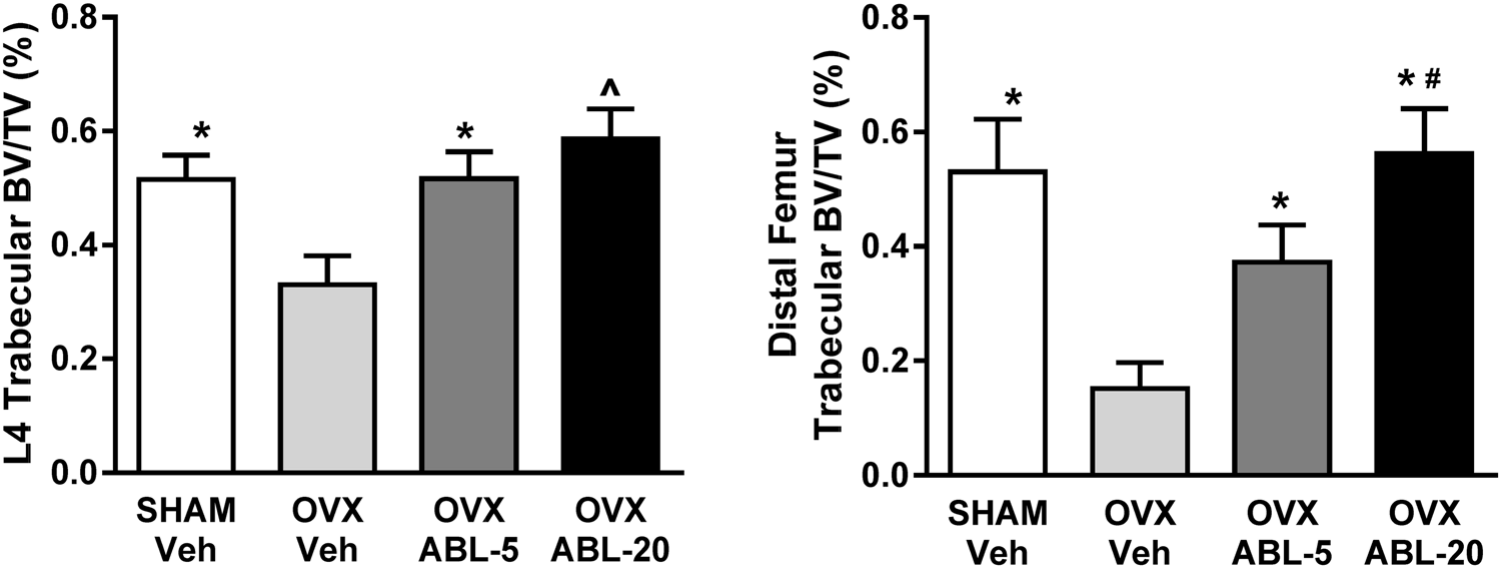
Effect of OVX and abaloparatide administration on trabecular bone volume fraction (BV/TV) of the L4 vertebral body (*left panel*) and the distal femoral metaphysis (*right panel*) as assessed by µCT. ABL-5, abaloparatide 5 µg/kg/day; ABL-20, abaloparatide 20 µg/kg/day. Data are mean ± SEM, *n* = 20–24 per group. *P < 0.001 versus OVX-Veh; ^^^ *P* < 0.001 versus all other groups;^ #^ *P* < 0.001 for ABL-20 versus ABL-5

Total femur aBMD increased significantly and dose-dependently in the OVX-ABL5 and OVX-ABL20 groups relative to baseline (by 21 and 27 %, respectively; *P* < 0.001, Fig. 1). The femur diaphysis exhibited similar increases in aBMD with abaloparatide relative to baseline values (Fig. 1). These gains in femur aBMD resulted in post-treatment aBMD values in the abaloparatide groups that significantly exceed those of OVX-Veh or Sham controls (both *P* < 0.001, Figs. 1, 2). Collectively, these data demonstrated marked gains in bone mass in response to abaloparatide treatment.

### Bone Microarchitecture and Volumetric BMD

Consistent with the aBMD measurements, µCT analyses indicated significant bone deterioration in the OVX-Veh group relative to Sham controls at the end of the treatment phase, particularly in trabecular compartments (Fig. 2; Tables 2, 3). Compared to Sham control rats, L4 of OVX-Veh rats had 42 % lower trabecular vBMD (Tb.vBMD) (Table 2, *P* < 0.001), 36 % lower trabecular BV/TV (Tb.BV/TV) (Fig. 2; Table 2, *P* < 0.001), significantly lower values for trabecular number (Tb.N), trabecular thickness (Tb.Th) and bone volume (BV), and greater trabecular spacing (Tb.Sp) (Table 2). At the distal femur, Tb.vBMD was 64 % lower and Tb.BV/TV was 71 % lower in OVX-Veh rats relative to Sham rats (Fig. 2; Table 3, *P* < 0.001), and Tb.N, Tb.Th and Conn.D were also lower in OVX-Veh rats versus Sham controls (Table 3). Cortical bone was also decreased by OVX, with cortical BV/TV (Ct.BV/TV) and cortical thickness (Ct.Th) significantly lower in the femur diaphysis of OVX-Veh rats compared with Sham controls (Table 3, *P* < 0.01 versus Sham).

**Table 2:** Effect of OVX and abaloparatide administration on trabecular bone in the L4 vertebral body as assessed by μCT

	SHAM vehicle	OVX vehicle	OVX abaloparatide	
			5 µg/kg	20 µg/kg
L4 lumbar spine				
vBMD (mg/mm^3^)	560 ± 35*	394 ± 51^§^	570 ± 46*	631 ± 50*^,§,^^
BV/TV (%)	51.5 ± 4.3*	33.0 ± 0.5^§^	51.7 ± 4.7*	58.6 ± 5.3*^,§,^^
Tb.Th (mm)	0.110 ± 0.01*	0.095 ± 0.00^§^	0.136 ± 0.01*^,§^	0.152 ± 0.01*^,§,^^
Tb.N (1/mm)	4.87 ± 0.28*	3.62 ± 0.48^§^	3.91 ± 0.30*^,§^	4.05 ± 0.27*^§^
Tb.Sp (mm)	0.181 ± 0.01*	0.268 ± 0.05^§^	0.219 ± 0.03*^,§^	0.201 ± 0.02*^,§,^^
Conn.D (1/mm^3^)	75.0 ± 12.9	68.3 ± 11.3	48.0 ± 5.4*^,§^	42.1 ± 7.2*^,§,^^
SMI	−1.82 ± 0.74*	0.29 ± 0.43^§^	−1.33 ± 0.56*	−2.23 ± 0.92*^,§,^^

Data are mean ± SD. *n* = 20–24 per treatment group

*vBMD* volumetric bone mineral density, *BV* bone volume, *TV* total volume, *Tb.Th* trabecular thickness, *Tb.N* trabecular number, *Tb.Sp* trabecular separation, *Conn.D* connectivity density, *SMI* structure model index

*P < 0.05 versus OVX-vehicle group; ^§^ *P* < 0.05 versus Sham group; ^^^ *P* < 0.05 versus ABL 5 μg/kg group

**Table 3:** Effect of OVX and abaloparatide administration on distal femur trabecular architecture and femur diaphysis geometry, as assessed by μCT

	SHAM vehicle	OVX vehicle	OVX abaloparatide	
			5 µg/kg	20 µg/kg
Distal femur trabecular bone				
vBMD (mg/mm^3^)	575 ± 79*	199 ± 56^§^	421 ± 65*^,§^	596 ± 84*^,^^
BV/TV (%)	53.0 ± 9.2*	15.2 ± 4.5^§^	37.2 ± 6.5*^,§^	56.2 ± 7.9*^,^^
Tb.Th (mm)	0.119 ± 0.02*	0.087 ± 0.01^§^	0.130 ± 0.01*^,§^	0.186 ± 0.03*^,§,^^
Tb.N (1/mm)	5.74 ± 0.62*	1.66 ± 0.59^§^	2.43 ± 0.66*^,§^	3.01 ± 0.51*^,§,^^
Tb.Sp (mm)	0.148 ± 0.03*	0.715 ± 0.28^§^	0.494 ± 0.17*^,§^	0.399 ± 0.11*^,§,^^
Conn.D (1/mm^3^)	115.9 ± 19.5*	42.7 ± 12.8^§^	53.1 ± 10.6*^,§^	34.7 ± 9.0*^,§,^^
SMI	−1.67 ± 2.31*	1.58 ± 0.17^§^	−0.39 ± 0.46*^,§^	−3.26 ± 1.73*^,§^
Femur diaphysis cortical bone				
Ct.Ar/Tt.Ar (%)	67.3 ± 3*	66.3 ± 2^§^	66.8 ± 4^§^	70.0 ± 3*^,§^
Ct.Ar (mm^2^)	3.22 ± 0.17	3.31 ± 0.22	3.60 ± 0.22*^,§^	3.67 ± 0.33*^,§^
Tt.Ar (mm^2^)	4.80 ± 0.34	4.99 ± 0.36	5.41 ± 0.52*^,§^	5.26 ± 0.59^§^
M.Ar (mm^2^)	1.57 ± 0.23	1.68 ± 0.20	1.81 ± 0.35	1.59 ± 0.31
Ct.Th (mm)	0.616 ± 0.09*	0.674 ± 0.04^§^	0.703 ± 0.04*^,§^	0.723 ± 0.05*^,§^

Data are mean ± SD. *n* = 20–24 per treatment group

*vBMD* volumetric bone mineral density, *BV* bone volume, *TV* total volume, *Tb.Th* trabecular thickness, *Tb.N* trabecular number, *Tb.Sp* trabecular separation, *Conn.D* connectivity density, *SMI* structure model index, *Ct.Ar* cortical area, *Tt.Ar* total area, *M.Ar* marrow area

* *P* < 0.05 versus OVX-vehicle group; ^§^ *P* < 0.05 versus Sham group; ^^^ *P* < 0.05 versus ABL 5 μg/kg group

Six-week treatment with abaloparatide improved vBMD and bone microarchitectural properties in OVX rats and fully inhibited OVX-induced bone loss, improving cortical and trabecular bone parameters to levels at or above the OVX-Veh and Sham controls. Specifically, L4 from the OVX-ABL20 group exhibited significantly higher Tb.vBMD and Tb.BV/TV compared to OVX-Veh animals (both +77 %, *P* < 0.001; Fig. 2; Table 2) and Sham-Veh animals (+13 and +14 %, respectively, *P* < 0.001; Fig. 2; Table 2). The OVX-ABL5 group had 45 % greater L4 Tb.vBMD and 56 % greater L4 Tb.BV/TV relative to OVX-Veh treatment (both *P* < 0.001; Table 2). Both doses of abaloparatide were also associated with improved trabecular architecture in L4, including increased trabecular number and thickness, reduced trabecular spacing and decreased SMI values indicating a more plate-like arrangement (Table 2).

At the distal femur, the OVX-ABL5 and OVX-ABL 20 groups exhibited Tb.vBMD and Tb.BV/TV values that were 2.1- to 3.7-fold greater than those of OVX-Veh controls (*P* < 0.001; Fig. 2; Table 3). The distal femur of both abaloparatide groups also exhibited significant increases in Tb.Th and Tb.N, along with lower Tb.Sp and SMI, indicative of a more robust plate-like trabecular architecture relative to OVX-Veh controls (Table 3). Evaluation of cortical responses to treatments in the femur mid-shaft indicated that the OVX-ABL20 group had significantly greater Ct.Ar/Tt.Ar compared with the OVX-Veh group (*P* < 0.05, Table 3) and Sham controls (*P* < 0.001, Fig. 2; Table 3). Cortical thickness was also significantly greater in the OVX-ABL20 group relative to OVX-Veh controls (*P* < 0.05, Table 3).

### Vertebral and Femoral Bone Strength

OVX resulted in compromised structural strength of L4, as shown by significantly lower values for maximum load and energy in OVX-Veh rats compared to Sham controls (*P* < 0.01, Table 4). These bone strength deficits were fully reversed by abaloparatide, with the OVX-ABL5 and OVX-ABL20 groups exhibiting maximum load and energy values significantly exceeding those from the OVX-Veh and Sham control groups (Table 4). In addition to these structural strength parameters, vertebral material (i.e., tissue-level) properties were explored by adjusting L4 maximum load values for bone volume and for bone density. The bone volume adjustment was accomplished by dividing L4 maximum load by L4 specimen height, cross-sectional area and micro-CT-derived BV/TV. This estimate of ultimate strength indicated no significant differences between any groups (Table 4). Bone density adjustment was accomplished by dividing L4 maximum load by micro-CT-derived L4 vBMD. The resulting BMD-adjusted maximum load values were all statistically similar, with group mean values (±SEM) of 0.472 ± 0.006 N/g/cm^3^ for Sham-Veh, 0.494 ± 0.010 N/g/cm^3^ for OVX-Veh, 0.559 ± 0.006 N/g/cm^3^ for OVX-ABL-5 and 0.532 ± 0.006 N/g/cm^3^ for OVX-ABL-20.

**Table 4:** Effect of OVX and abaloparatide administration on L4 lumbar spine biomechanical properties

	SHAM vehicle	OVX vehicle	OVX abaloparatide	
			5 µg/kg	20 µg/kg
Vertebral compression				
Maximum load (N)	265 ± 81*	190 ± 71^§^	323 ± 68*^,§^	336 ± 76*^,§^
Stiffness (N/mm)	2032 ± 913	1795 ± 894	1872 ± 1037	1845 ± 954
Energy (mJ)	35 ± 16*	22 ± 12^§^	62 ± 38*^,§^	64 ± 29*^,§^
Adjusted ult. strength (N/mm^2^/BV/TV)	65.6 ± 0.7	74.7 ± 1.3	77.9 ± 0.8	70.17 ± 0.7

Data are mean ± SD. *n* = 20–24 per treatment group

*Ult* ultimate

* *P* < 0.05 versus OVX-vehicle group; ^§^ *P* < 0.05 versus Sham group

Femur diaphysis three-point bending tests were also conducted to assess effects of abaloparatide on cortical bone strength. Consistent with other OVX rat studies [27], femur diaphysis structural strength parameters tended to be higher in OVX-Veh rats compared with Sham-Veh rats, with maximum load and energy values that were 8 and 27 % higher, respectively, than Sham controls (both *P* < 0.05, Table 5). The OVX-ABL5 and OVX-ABL20 groups had significantly improved structural strength of the femur diaphysis, with maximum load and stiffness values exceeding those of the OVX-Veh control group by 9–12 % (<0.05, Table 5). Maximum load, stiffness and energy values in the abaloparatide groups also significantly exceeded Sham control group values (all *P* < 0.01, Table 5). Apparent material properties of the femur diaphysis were modestly influenced by OVX and by abaloparatide. Toughness was significantly greater in OVX-Veh versus Sham controls, and ultimate strength and toughness were significantly greater in OVX-ABL5 and OVX-ABL20 groups relative to Sham controls (all *P* < 0.05, Table 5). Axial area moment of inertia of the femur diaphysis was approximately 17 % greater in each OVX-ABL group relative to sham controls (*P* < 0.001, Table 5).

**Table 5:** Effect of OVX and abaloparatide administration on femur biomechanical properties

	SHAM vehicle	OVX vehicle	OVX abaloparatide	
			5 µg/kg	20 µg/kg
Femur shaft bending strength				
Maximum load (N)	188 ± 14*	204 ± 21^§^	223 ± 16*^,§^	224 ± 25*^,§^
Stiffness (N/mm)	771 ± 105	779 ± 133	874 ± 120*^,§^	872 ± 127*^,§^
Energy (mJ)	56 ± 16*	71 ± 19^§^	78 ± 17^§^	76 ± 20^§^
Ult. strength (N/mm^2^)	173 ± 16	177 ± 15	185 ± 18^§^	184 ± 19^§^
Elastic modulus (MPa)	7479 ± 1113	7100 ± 1173	7381 ± 1502	7449 ± 1480
Toughness (MJ/m^3^)	4.9 ± 1.5*	5.8 ± 1.4^§^	6.3 ± 1.3^§^	6.0 ± 1.4^§^
AP diameter (mm)	3.1 ± 0.1	3.1 ± 0.1	3.2 ± 0.1*^,§^	3.2 ± 0.2
AAMI (mm^4^)	5.9 ± 0.7	6.3 ± 0.8	6.9 ± 1.0*^,§^	6.9 ± 1.3^§^
Femur neck cantilever compression strength				
Maximum load (N)	100 ± 13	93 ± 15	123 ± 25*^,§^	116 ± 20*^,§^
Stiffness (N/mm)	216 ± 50	189 ± 55	226 ± 65	198 ± 56
Energy (mJ)	31 ± 10	36 ± 11	46 ± 25^§^	46 ± 14*^,§^

Data are mean ± SD. *n* = 20–24 per treatment group

*Ult.* ultimate, *AP* antero-posterior, *AAMI* axial area moment of inertia

* *P* < 0.05 versus OVX-vehicle group; ^§^ *P* < 0.05 versus Sham group

Cantilever compression of the femoral neck showed that the maximum load tolerated was 7 % lower in OVX-Veh-treated rats relative to Sham controls (*P* < 0.01 versus Sham, Table 5). The OVX-ABL5 and OVX-ABL20 groups exhibited increased femur neck strength, with maximum load (23 and 16 %, respectively, *P* < 0.01, Table 5), and energy (48 %, *P* < 0.05) higher than OVX-Veh control.

### Bone Mass–Strength Relationships

**Fig. 3:**
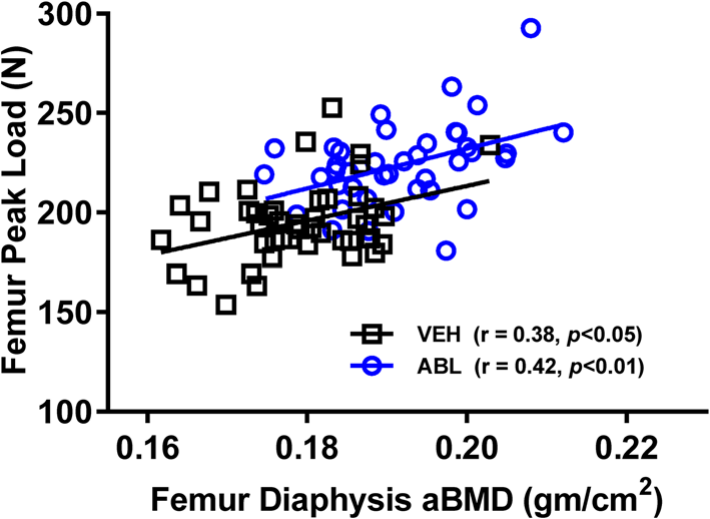
Relationship between bone mass and bone strength for the femur diaphysis. Femur aBMD was assessed at the femur diaphysis by DXA, and peak load was determined by three-point bending. The OVX-Veh and Sham-Veh animals were combined into one Veh group, and the ABL-5 and ABL-20 animals were combined into one ABL group. For both groups, regression line slopes were significantly different from zero, and there were no significant differences between the two groups for slope or for *X* or *Y* intercepts

Relationships between femur diaphysis bone mass and strength were assessed for by linear regression analysis. For these analyses the OVX-Veh and Sham-Veh groups were combined into a single Veh group, and the ABL-5 and ABL-25 groups were combined into a single ABL group. Regression lines for the Veh and ABL groups had slopes that differed significantly from zero (*r* = 0.38 and 0.42, respectively, both *P* < 0.05) and did not differ from each other with regard to slope or X or Y intercepts (Fig. 3).

## Discussion

Abaloparatide is a novel peptide activator of the PTH1 receptor signaling pathway that is currently in late-stage clinical development for the treatment of postmenopausal women with osteoporosis. The primary goal of osteoporosis therapies is to reduce fracture risk by improving bone strength, and the gold-standard approach for assessing treatment effects on bone strength is destructive ex vivo biomechanical testing. The primary objective of this study was to combine destructive bone strength testing with high-resolution CT imaging in order to assess the effects of a 6-week regimen of daily abaloparatide injections in adult OVX rats. We hypothesized that abaloparatide treatment would reverse bone loss and the deterioration of bone mechanical properties associated with OVX-induced osteopenia by promoting gains in bone mass and restoration of bone microarchitecture. The current results, the first that describe the effects of abaloparatide on bone strength, indicate that treatment with abaloparatide fully reversed OVX-induced bone loss and increased bone strength at the lumbar spine, femur diaphysis and femur neck of OVX rats. In many cases, bone mass and strength parameters in the OVX-ABL groups exceeded those of ovary-intact sham controls, which further highlights the robust efficacy potential of abaloparatide. These observations of marked gains in bone mass following treatment with ABL in osteopenic OVX rats are consistent with the robust BMD gains seen in a Phase 2 clinical trial with abaloparatide treatment in postmenopausal woman with osteoporosis [25].

Abaloparatide administration to osteopenic OVX rats fully and dose-dependently restored aBMD at the lumbar spine and femur by 6 weeks, indicating a rapid onset of effects in cortical and cancellous compartments. Gains in spine and femur aBMD appear to be mediated primarily by increases in trabecular and cortical bone volume. Improvements in cortical bone were evident from increased aBMD of the mid-femur diaphysis, a purely cortical site, and from µCT-derived evidence for increases in cortical bone volume and cortical thickness. Improvements in trabecular bone with abaloparatide were revealed by µCT assessments of L4 and the distal femur metaphysis, which demonstrated increases in trabecular bone volume and vBMD, and improved trabecular microarchitecture. Microarchitectural improvements with abaloparatide included more numerous and thicker trabeculae, with reduced spacing and a more plate-like arrangement, each of which may have contributed to the observed improvements in vertebral strength. High-dose abaloparatide was also associated with reduced trabecular connectivity density values for L4 and the distal femur, and analyses of human vertebrae suggest that reduced connectivity density may be associated with greater structural strength [28]. This seemingly paradoxical relationship does not indicate that reduced connectivity per se is biomechanically advantageous, but that trabecular structures with more connections can in some cases be less robust.

Abaloparatide-related gains in bone mass and improvements in bone microarchitecture parameters were associated with improved biomechanical parameters. Evidence that BMD gains with abaloparatide leads to improved bone strength in this preclinical osteoporosis model extends and complements recently reported clinical study results where abaloparatide treatment increased BMD in lumbar spine and total hip in a Phase 2 clinical study of postmenopausal women with osteoporosis [25]. BMD gains with abaloparatide were rapid in both studies, reversing the catabolic effects of 8 weeks of OVX-induced estrogen deficiency within 6 weeks in these rats, and significantly increasing lumbar spine and total hip BMD within 12 weeks in women with osteoporosis [25]. In the Phase 2 study, BMD gains with abaloparatide were greater than those observed with teriparatide [rhPTH(1-34)] at both the 12- and 24-week time points [25]. Data on the effect of abaloparatide on vertebral and nonvertebral fracture risk are starting to emerge from a Phase 3 clinical trial [29], which support the notion that rapid and substantial BMD gains with abaloparatide are accompanied by substantial fracture risk reduction.

The effects of abaloparatide treatment were seen in all regions of the femur, suggesting that the effect on BMD potentially includes positive effects on both the trabecular and cortical bone compartments. The pharmacological mechanisms underlying the BMD effects in the cortical bone observed with abaloparatide treatment are not entirely clear. Additional studies will be required to further evaluate the potential effects on both the trabecular and cortical compartments. The current study provides the first animal data indicating increased cortical BMD with abaloparatide, along with increases in trabecular bone microarchitecture parameters, which were associated with increased bone strength.

Destructive biomechanical assessments of bone strength revealed substantial improvements in structural strength in abaloparatide-treated animals. The effects of abaloparatide included greater maximum load for L4, the femur diaphysis and femur neck relative to either OVX-Veh or Sham controls. It may be noteworthy from a bone quality perspective that abaloparatide-induced increments in maximum load of L4 and the femur diaphysis were well aligned with the magnitude of gains in bone mass parameters measured at the same skeletal site, which suggests that bone matrix that accrues during abaloparatide therapy has good material properties. Femur BMD values were positively and similarly correlated with maximum load values in the two Veh control groups and in the two abaloparatide groups, suggesting that abaloparatide had no deleterious effects on bone quality. Bone quality was also assessed by adjusting structural strength parameters for bone geometry (femur diaphysis) or for specimen size and bone volume fraction (L4 vertebral body). By this approach, both abaloparatide groups showed similar material properties compared with OVX-Veh control groups for both skeletal sites, indicating maintenance of material strength properties. Based on these findings, it is reasonable to conclude that increased bone structural strength with abaloparatide is largely a function of increased bone mass, density and volume. These initial bone quality assessments will be followed by additional studies conducted with longer treatment durations to better assess the impacts of abaloparatide on bone strength and bone quality.

The molecular mechanisms by which abaloparatide exerts its effects on bone are not fully understood but may be related to confirmation-selective binding to the PTH1 receptor relative to other ligands including PTH and PTHrP. PTH and PTHrP share some sequence homology and may have arisen by duplication of a common ancestral gene, but each plays a distinct role in bone physiology. PTH, which is secreted by the parathyroid glands, acts in a classical endocrine manner to promote osteoclastic bone resorption and calcium mobilization. In contrast, PTHrP functions as a paracrine regulator of bone formation. Despite these differences, PTH and PTHrP both increase intracellular cAMP concentrations by activating the same PTHR1, a G protein-coupled receptor (GPCR). However, continuous administration of PTH leads to bone resorption over formation, whereas continuous PTHrP administration preferentially stimulates formation [30, 31]. Recent studies have provided a basis for the divergent actions of PTH and PTHrP in bone. Specifically, PTHrP activity at PTHR1 is restricted to the cell surface and yields a brief intracellular cAMP burst, whereas the receptor conformation associated with PTH stabilizes its binding to the receptor and its coupled G protein and moves to internalized compartments of the cell, leading to persistent cAMP generation [17, 19, 32, 33]. The significance of ligands that form more stable complexes and more cAMP responses is a more catabolic response resulting in elevated blood calcium levels [18]. In contrast, ligands such as PTHrP transiently produce cAMP and mobilize calcium, yet results in greater anabolic action than PTH. Consistent with these reports, a recent study evaluated the binding of abaloparatide to two distinct PTHR1 conformations. The findings suggested that the enhanced bone anabolic activity seen with abaloparatide treatment may arise from a more selective binding to the R0 PTHR1 than the RG conformation, compared to PTH (1-34), a long-acting PTH (LA-PTH) analog or PTHrP [34]. Further studies are required to elucidate the specific molecular mechanisms of abaloparatide that cause increased anabolic activity.

This study has several strengths, including a robust number of animals that provided good statistical power for the ex vivo assessments of bone architecture and strength. Many novel bone therapeutics were initially tested in healthy animals with normal bone mass, whereas this index pharmacology report for abaloparatide involved ovariectomized rats with established osteopenia, which represents a reasonably high hurdle for efficacy and proof of concept for osteoporosis therapy. The study also involved bone strength assessments at multiple skeletal sites that correspond to areas at increased risk of fracture in postmenopausal women. The study also has several limitations, some of which relate to the study goal as an initial pharmacology assessment, including the use of young growing animals, the absence of data for histomorphometry and for bone turnover markers. The study also lacked µCT data on the proximal femur including the femur neck, and therefore, the compartment-specific structural changes associated with improved femur neck strength in the abaloparatide groups remain unknown. Future longer-term studies will address these questions.

In summary, 6 weeks of abaloparatide administration to OVX osteopenic rats increased bone mass and microarchitecture parameters and increased bone strength. Abaloparatide was well tolerated, and there was no evidence for impairment of bone quality or reductions in bone material properties. The overall data indicate that abaloparatide administration results in rapid and robust gains in bone mass and bone strength, and support the continued investigation of abaloparatide as potential therapy for the treatment of postmenopausal women with osteoporosis.
